# A refined protocol for the isolation and monoculture of primary mouse renal peritubular endothelial cells

**DOI:** 10.3389/fcvm.2023.1114726

**Published:** 2023-02-09

**Authors:** Austin D. Thompson, Jaroslav Janda, Rick G. Schnellmann

**Affiliations:** ^1^Department of Pharmacology and Toxicology, College of Pharmacy, Bio5 Institute, The University of Arizona, Tucson, AZ, United States; ^2^Southwest Environmental Health Sciences Center, Tucson, AZ, United States; ^3^Southern Arizona Veterans Affairs (VA) Health Care System, Tucson, AZ, United States

**Keywords:** acute kidney injury, peritubular capillaries, microvasculature, primary endothelial cell isolation, cardiorenal syndrome (CRS), renal endothelium, AKI progression

## Abstract

During an episode of acute kidney injury (AKI), a sudden and rapid decline in renal function is often accompanied by a persistent reduction in mitochondrial function, microvasculature dysfunction/rarefaction, and tubular epithelial injury/necrosis. Additionally, patients who have experienced an AKI are at an elevated risk of developing other progressive renal, cardiovascular, and cardiorenal related diseases. While restoration of the microvasculature is imperative for oxygen and nutrient delivery/transport during proper renal repair processes, the mechanism(s) by which neovascularization and/or inhibition of microvascular dysfunction improves renal recovery remain understudied. Interestingly, pharmacological stimulation of mitochondrial biogenesis (MB) post-AKI has been shown to restore mitochondrial and renal function in mice. Thus, targeting MB pathways in microvasculature endothelial cell (MV-EC) may provide a novel strategy to improve renal vascular function and repair processes post-AKI. However, limitations to studying such mechanisms include a lack of commercially available primary renal peritubular MV-ECs, the variability in both purity and outgrowth of primary renal MV-EC in monoculture, the tendency of primary renal MV-ECs to undergo phenotypic loss in primary monoculture, and a limited quantity of published protocols to obtain primary renal peritubular MV-ECs. Thus, we focused on refining the isolation and phenotypic retention of mouse renal peritubular endothelial cells (MRPEC) for future physiological and pharmacological based studies. Here, we present a refined isolation method that augments the purity, outgrowth, and phenotypic retention of primary MRPEC monocultures by utilizing a collagenase type I enzymatic digestion, CD326+ (EPCAM) magnetic microbead epithelial cell depletion, and two CD146+ (MCAM) magnetic microbead purification cycles to achieve a monoculture MRPEC purity of ≅ 91–99% by all markers evaluated.

## 1. Introduction

Acute kidney injury (AKI) is characterized as an episode of sudden and rapid decline in renal function and is often accompanied by a persistent reduction in mitochondrial function, renal microvasculature endothelial cell (MV-EC) dysfunction/rarefaction, and tubular injury/necrosis (1). Common causes of AKI include sepsis, physical trauma, xenobiotic/toxicant exposure, ischemia/reperfusion (I/R), severe dehydration, and major surgery (2). AKI continues to be an immense public health concern, as there remains no effective FDA-approved treatment options, high patient mortality rates (∼3–18%), and an enormous financial burden on health care systems (∼$4.7–$24 billion annually) (3, 4). Moreover, patients who have been diagnosed with an AKI are at an elevated risk of developing recurrent-AKI, sepsis, renal cancers, chronic kidney disease, end-stage renal disease, and cardiovascular diseases (CVDs) (2, 5–7). An important underlying contributor to AKI, and the subsequent development of other progressive kidney and cardiovascular related diseases, is renal MV-EC dysfunction/rarefaction (1, 2, 6–8).

Renal MV-ECs are positioned within the capillary beds of the kidney and are imperative for the maintenance of various biological processes such as coagulation, angiogenesis, inflammation, vascular permeability, and solute/lipid transport (6, 8, 9). Furthermore, MV-ECs express a unique metabolic profile compared to most other cell types, as they primarily utilize aerobic glycolysis to produce most of their adenosine 5′-triphosphate (ATP) (10). While the exact causes of renal injury following AKI remain unclear, tubular and MV-EC dysfunction along with excessive inflammatory immune cell infiltration and activation are known to be major contributing factors (8, 10). Additionally, the generation of numerous reactive oxygen species (ROS) and the induction of various pro-inflammatory signaling pathways following AKI exacerbates microvascular permeability and reduces renal vascular integrity (8, 11). Importantly, ROS can induce direct endothelial cell (EC) damage while pro-inflammatory responses activate ECs to facilitate the transmigration of leukocytes into extravascular tissue, further increasing microvascular permeability and dysfunction (11).

Renal MV-EC rarefaction has been previously observed in both human and animal studies of AKI (12). This MV-EC rarefaction exacerbates renal injury and increases the likelihood of AKI recurrence (∼20% of all AKI patients), as the renal microvasculature is essential for oxygen and nutrient delivery to extravascular tissue during proper renal repair processes (13). Various *in vivo* rodent studies have revealed that renal peritubular endothelial cells (RPEC) experience a high percentage (∼25–45%) of microvascular rarefaction and undergo endothelial-to-mesenchymal-transition post-AKI (∼10–15%) (14). Furthermore, AKI results in a persistent reduction in mitochondrial function, number, and cellular energetics (15). Conversely, pharmacological stimulation of mitochondrial biogenesis (MB) has been reported to enhance glomerular MV-EC function and promote functional renal recovery proceeding an AKI (16, 17). However, minimal AKI research has focused on the cellular pathways that govern proper RPEC repair mechanisms or how stimulation of MB contributes to these processes (18). This is in part due to the lack of commercially available RPECs, variability in the purity of primary isolated RPECs, and the difficulty of isolating/maintaining these cells in primary monoculture, as they are prone to undergo phenotypic loss (19). Thus, we developed a refined protocol for the isolation and monoculture of mouse renal peritubular endothelial cells (MRPEC) to address these issues and obtain MRPECs of suitable quality for downstream *in vitro* physiologically and pharmacological microvasculature experiments.

## 2. Materials and equipment

The composition, concentrations, technical notes, and supplier information for all components utilized to generate buffers and solutions within this protocol can be found in Table 1. Furthermore, all remain materials, reagents, antibodies, and equipment used within this protocol along with their respective concentrations, technical notes, and supplier information can be found in Table 2.

**TABLE 1:** Compositions and components of buffers and solutions for primary MRPEC isolation.

Materials/reagents	Notes	Concentrations	Source	Product ID
Part A: Formulation of collagenase type I enzymatic tissue digestion buffer*				
DMEM/F-12, GlutaMAX supplement, Media	–	Used as supplied by manufacturer	Gibco	10565018
Collagenase, Type I, powder	As manufacturer lot numbers vary in enzymatic activity, adjust quantity to enzymatic activity units	∼1.25 mg/ml (∼350 enzymatic activity units)	Gibco	17100-017
DNAse-1	DNase I recombinant, RNase-free	100 U/ml	Roche	4716728001
Bovine serum albumin (BSA)	Use BSA heat shock fraction-V, protease free, fatty acid free, essentially globulin free, pH 7, ≥98%	1 mg/ml	Sigma-Aldrich	A7030-50G
Part B: Formulation of Percoll-Plus solution*				
DMEM/F-12, HEPES, Media	–	12.5/25 ml (v/v)	Gibco	11-330-057
Percoll PLUS Centrifugation Media	Used for gradient centrifugation steps	11.25/25 ml (45% v/v)	Cytiva	17544502
20× phosphate buffered saline, powder, pH 7.4	Prepare a 20× PBS stock solution by adding one packet of PBS powder to 50 ml of filtered ultrapure water	562.5 μl/25 ml (v/v)	Sigma-Aldrich	P3813-5X10PAK
Ultra-Pure DNase/RNase-Free Distilled Water	–	687.5 μl/25 ml (v/v)	Invitrogen	10977023
Part C: Formulation of magnetic microbead solution*				
1× phosphate buffer saline (PBS), pH 7.4	Without Ca^2+^ and Mg^2+^ (PBS^–/–^)	400 ml	Gibco	10010049
Ethylenediaminetetraacetic acid (EDTA)	A 500 mM stock solution was initially prepared and then diluted to a final concentration of 2 mM	2 mM [1.6 ml (500 mM stock) / 400 ml]	Sigma-Aldrich	E9884-1Kg
Bovine serum albumin (BSA)	Use BSA heat shock fraction, protease free, fatty acid free, essentially globulin free, pH 7, ≥98%	2 g (0.5% w/v)	Sigma-Aldrich	A7030-50G
Part D: Formulation of MV-EC specific cell culture media*				
EGM™-2MV (Microvasculature Endothelial Cell Growth Medium-2) BulletKit™	Product contains: EBM™-2 Basal Endothelial Cell Medium and EGM™-2MV SingleQuots™ Supplement Pack	Used/prepared as supplied by manufacturer	Lonza	CC-3202
Heparin sodium solution	Heparin sodium USP at 1,000 U/ml	0.75 U/ml (375 μl/500 ml of media)	Mylan	NDIC: 67457-385-99
Mouse VEGF-164 Recombinant Protein	Lyophilized from a 0.2 μm filtered solution in PBS	50 ng/ml (25 μg/500 ml of media)	R&D Systems	493MV025CF
Part E: Formulation of FACS buffer*				
1× phosphate buffer saline, pH 7.4	Without Ca^2+^ and Mg^2+^ (PBS^–/–^)	400 ml	Gibco	10010049
Ethylenediaminetetraacetic acid (EDTA)	A 500 mM stock solution was initially prepared and then diluted to a final concentration of 2 mM	2 mM (1.6 ml of 500 mM stock/400 ml)	Sigma-Aldrich	E9884-1Kg
100% fetal calf serum	–	4 /400 ml (1% v/v)	Lonza	CC-3202

*As the volume of each buffer required for this protocol will vary depending on the number of mice being utilized, scale-up/down the final volume and concentrations proportionally based on this table.

**TABLE 2:** Reagents and resources list for MRPEC isolation.

Antibodies/reagents	Host/clonality	Concentrations	Source	Product ID
**Part A: Immunofluorescence staining and flow cytometry analysis antibodies/nuclear reagents with specific concentrations used**				
**Primary/primary-conjugated antibodies**				
Anti-VE-cadherin antibody (EPR18229)]	Recombinant Rabbit anti-Mouse Monoclonal; accession #P33151	1:1,000	Abcam	ab205336
Anti-PLVAP/PV-1 antibody (MECA-32)	Recombinant Rat anti-PLVAP/PV-1 Monoclonal; accession #Q9BX97	1:500	Abcam	ab27853
Anti-isolectin GS-IB4 (GSL-1) AF488-conjugated antibody	Isolectin GS-IB4, *Griffonia simplicifolia*, Alexa Fluor 488 Conjugated	20 μg/ml	Invitrogen	I21411
Anti-CD31/PECAM-1 antibody	Goat anti-Mouse/Rat Polyclonal; accession #Q08481	1:100	Novus	AF3628
Anti-EHD3 Antibody (RR-L)	Mouse anti-Mouse monoclonal IgG1-κ EHD3 antibody (RR-L)	1:250	Santa Cruz	sc-100723
Recombinant anti-S100A4 antibody [EPR14639(2)]	Recombinant Rabbit anti-Mouse S100A4 Monoclonal	1:250	Abcam	ab197896
Recombinant anti-NG2 antibody (EPR23976-145)	Recombinant Rabbit anti-Mouse NG2 Monoclonal	1:100	Abcam	ab275024
Recombinant anti-EpCAM antibody (EPR20532-222)	Recombinant Rabbit anti-Mouse EPCAM Monoclonal	1:100	Abcam	ab213500
**Secondary antibodies**				
**Donkey anti-Rabbit Alexa Flour 488+ (**used for VE-cadherin IF secondary staining in Figures 4A, B)	Donkey anti-Rabbit IgG (H + L) Highly Cross-Adsorbed Alexa Fluor Plus 488; RRID#AB_2762833	1:1,000	Invitrogen	A32790
**Donkey anti-Goat Alexa Flour 555+ (**used for PECAM1 (CD31) IF secondary staining in Figure 4A)	Donkey anti-Goat IgG (H + L) Highly Cross-Adsorbed Alexa Fluor Plus 555; RRID#AB_2762839	1:500	Invitrogen	A32816
**Donkey anti-Rat Alexa Flour 555+ (**used for PLVAP (PV-1) IF secondary staining in Figure 4B)	Donkey anti-Rat IgG (H + L) Highly Cross-Adsorbed Alexa Fluor Plus 555	1:500	Invitrogen	A48270
**Goat anti-Mouse Alexa Fluor 488 (**used for EHD3 IF secondary staining in Supplementary Figure 2A)	Goat anti-Mouse IgG (H + L) Cross-Adsorbed Secondary Antibody, Alexa Fluor 488	1:500	Invitrogen	A-11001
**Nuclear staining**				
Ibidi Mounting Medium with DAPI	–	300 μl of mounting media/imaging dish	Ibidi	50011
4′,6-Diamidino-2-phenylindole, dihydrochloride (DAPI) *FluoroPure grade*	–	Final diluted concentration: 300 nM	Invitrogen	D21490
**Flow cytometry antibodies**				
CD146 (LSEC) antibody, PE, REAfinity	Recombinant human IgG1; Human anti-Mouse Monoclonal (REA1064)	1:50	Miltenyi Biotec	130-118-407
CD144 (VE-cadherin) antibody, APC, REAfinity	Recombinant human IgG1; Human anti-Mouse Monoclonal (REA225)	1:50	Miltenyi Biotec	130-102-738
CD309 (VEGFR-2) antibody, APC, REAfinity	Recombinant human IgG1; Human anti-Mouse Monoclonal (REA1116)	1:50	Miltenyi Biotec	130-119-434
CD31 antibody, VioBright B515, REAfinity	Recombinant human IgG1; Human anti-Mouse Monoclonal (REA784)	1:50	Miltenyi Biotec	130-111-544
REA control antibody (S), PE, REAfinity	Recombinant human IgG1; Human anti-Mouse Monoclonal (REA293)	1:50	Miltenyi Biotec	130-113-438
REA control antibody (S), APC, REAfinity	Recombinant human IgG1; Human anti-Mouse Monoclonal (REA293)	1:50	Miltenyi Biotec	130-113-434
REA control antibody (S), VioBright B515, REAfinity	Recombinant human IgG1; Human anti-Mouse Monoclonal (REA293)	1:50	Miltenyi Biotec	130-113-445
CD31 (PECAM-1) monoclonal antibody (390), APC, eBioscience	CD31 (PECAM-1) Rat IgG2a kappa; Rat anti-Mouse Monoclonal Antibody (390)	1:50	eBioscience	17-0311-82
Rat IgG2a kappa isotype control (eBR2a), APC, eBioscience™	Rat IgG2a kappa Isotype Control; Rat anti-Mouse Monoclonal (eBR2a)	1:50	eBioscience	17-4321-81
**Part B: All other materials/reagents**				
Digitonin	Used for cell permeabilization (PLVAP and GSL-1 IF-staining only) at a final concentration of 10 μg/ml (w/v) in 1× DPBS^+/+^	10 μg/ml (w/v)	Abcam	ab141501
25G ×0.75 in. BD Vacutainer^®^ Safety-Lok	Remove blood collection tube/needle and instead attach the 25G needle w/tubing to a 50 ml syringe	–	BD	BD-367285
BD Luer-Lok Syringe sterile, single use, 50 ml	–	–	BD	BD-309653
TC20 Automated Cell Counter	–	–	Bio-Rad	1450102
5 ml Round Bottom Polystyrene Test Tube, with Cell Strainer Snap Cap	–	–	Corning	352235
Disposable Sterile Bottle-Top Filters with 0.22 μm Membrane 45 mm	–	–	Corning	431118
Disposable Sterile Bottle-Top Filters with 0.22 μm Membrane 33 mm	–	–	Corning	431117
Primaria 35 mm Cell Culture Dishes	–	–	Corning	353801
Matrigel basement membrane matrix, growth factor reduced, LDEV-free	–	–	Corning	354230
Polystyrene Serological Pipets, 5 ml	Sterile, single wrapped RNase-/DNase-free and BSE/TSE-free	–	Corning	356543
Polystyrene Serological Pipets, 10 ml	Sterile, single wrapped RNase-/DNase-free and BSE/TSE-free	–	Corning	356551
Polystyrene Serological Pipets, 25 ml	Sterile, single wrapped RNase-/DNase-free and BSE/TSE-free	–	Corning	356525
Cytiva Percoll™ PLUS Centrifugation Media	–	–	Cytiva	17544502
Easy Reader 15 ml Conical Polypropylene Centrifuge Tubes	–	–	Fisherbrand	05-539-4
Easy Reader 50 ml Conical Polypropylene Centrifuge Tubes	–	–	Fisherbrand	05-539-13
Sterile Cell Strainer 40 μm	–	–	Fisherbrand	22-363-547
HBSS, calcium, magnesium, no phenol red	–	–	Gibco	14025
DPBS, calcium, magnesium, glucose, pyruvate	–	–	Gibco	14287072
Penicillin-Streptomycin (10,000 U/ml)	–	1% of final volume when indicated	Gibco	15140-122
Polypropylene Graduated Microcentrifuge Tube with Snap Cap, 1.5 ml, Natural	–	–	Globe Scientific	111558
Advanced TC Treated Sterile Cell Culture Multi-Well Plates	–	–	Greiner Bio-One	662960
μ-Dish 35 mm, high, ibiTreat – Tissue Culture Treated Polymer Coverslip	–	–	Ibidi	81156
Image-iT Fixative Solution (4% formaldehyde, methanol-free)	Used in immunofluorescence experiments for cell fixation step	–	Invitrogen	R37814
BlockAid Blocking Solution	Used for cell blocking steps during immunofluorescence experiments	–	Invitrogen	B10710
Image-iT FX Signal Enhancer ReadyProbes™ Reagent	Used for cell blocking steps during immunofluorescence experiments	–	Invitrogen	R37107
LS Magnetic Columns	–	–	Miltenyi	130-042-401
CD146 (LSEC) MicroBeads mouse	Mixed end-over-end on microcentrifuge rotator in 4c	1:35	Miltenyi	130-092-007
CD326 (EPCAM) MicroBeads, mouse	Mixed end-over-end on microcentrifuge rotator in 4c	1:30	Miltenyi	130-105-958
MACS SmartStrainers (70 μm)	Used for cell straining prior to accutase digestion steps	–	Miltenyi	130-098-462
Pre-Separation Filters (70 μm)	Used with LS columns and MACS purification steps	–	Miltenyi	130-095-823
FcR Blocking Reagent, mouse	Used to block FcR on cells to reduce possibility of non-specific binding of magnetic microbeads	1:50	Miltenyi	130-092-575
gentleMACS Dissociator	Used for tissue digestion steps	–	Miltenyi	130-093-235
gentleMACS C Tubes	–	–	Miltenyi	130-093-237
Fibronectin Solution	Human Plasma Fibronectin	10 μg/ml/dish	PromoCell	C-43060
Fetal bovine serum	–	See text for more details (5–10% v/v)	Lonza	CC-3202
Triton X-100	Used for cell permeabilization at a final concentration of 0.1% (v/v) in 1× DPBS^+/+^	0.1% (v/v)	Sigma	T8787-100ML
Millex GP syringe filter unit, 0.22 μm, sterile	–	–	Sigma-Aldrich	SLGP033RS
Accutase^®^ solution	Used for second round of enzymatic digestion and removing cells from culture dishes	–	Sigma-Aldrich	A6964-100ML
Samco General Purpose Transfer Pipettes, Standard Bulb, 7.7 ml, Ind. Pack.	–	–	Thermo Fisher	202-1SPK
TIPONE FILTER TIPS (1000 XL Graduated 10 Rack of 96)	–	–	USA Scientific	1122-1830
TIPONE FILTER TIPS (200 Graduated 10 Rack of 96)	–	–	USA Scientific	1122-8810
TIPONE FILTER TIPS (10 XL Graduated 10 Rack of 96)	–	–	USA Scientific	1122-1830

FluoroPure grade means ≥98% pure by manufacturer’s HPLC analysis.

## 3. Methods

### 3.1. Step-by-step protocol: Isolation of mouse renal peritubular endothelial cells (MRPEC)

1. Perfuse murine kidneys by whole body perfusion *via* transcardial perfusion method with a 25-gauge needle (BD) and 30 ml of, DPBS^–/–^ or PBS^–/–^ (Gibco) containing 100 U/ml of Heparin (Mylan).
2. Remove the perfused murine kidneys and decapsulate them.*
° (*This step can be done during murine kidney harvest or prior to step 4. within a sterile laminar flow cell culture hood).
3. Submerge isolated kidneys in ice-cold 1:1 DMEM/Ham’s F12 (DMEM/F12; Gibco) media^**^ on ice in a petri dish or 15 ml conical tube.^***^
° [^**^We used DMEM/F12-GlutaMAX media (Gibco), 10% FBS, and 1% Penicillin-G and Streptomycin (PS)].
° (^***^Or any sterile holding container, on ice, that allows kidney samples to be fully submerged).
4. Cut kidneys sagittally, into two halves, using sterile razor blades or surgical scalpel, pin the halves to a dissection dish pre-filled with HBSS^+/+^ and remove the kidney cortices.
5. Place all kidney cortices from one mouse into a GentleMACS C-Tube^∧^ (Miltenyi Biotec) and resuspend the tissue pieces in 2 ml of fresh pre-warmed (37°C) collagenase I digestion solution.^#^
° [^∧^Alternatively, one can substitute GentleMACS C-Tube used in step 5. with a Dounce homogenizer equipped with a type A “loose” fit pestle and perturb the kidney cortices with 10–15 gentle strokes until tissue pieces are dispersed into the suspension (be as gentle as possible and do not over homogenize the tissue pieces. Note, this substitution will add time to the overall protocol and might decrease the overall cell viability/yield)].
• Collagenase Type I solution recipe per one mouse.
• 10 ml of DMEM/F12 (Gibco).
•∼1.25 mg/ml of Collagenase I (Gibco).
• 1 mg/ml of BSA (Fraction V, free of impurities especially immunoglobulins/complement components) (Sigma).
• 100 U/ml of DNase 1 (Invitrogen).
° (^#^See Table 1 for the exact solution compositions, considerations, and suppliers).
° (Note, be sure to sterile filter the entire solution by passing it through a 0.22-μm sterile filter prior to use).
6. Place GentleMACS C-Tube(s), containing the murine cortex tissues fully submerged in 2 ml of collagenase I digestion solution, onto an automated GentleMACS-Dissociator (Miltenyi Biotec) and dissociate the tissue pieces by running the preset program “m_brain_01.”
7. Remove the GentleMACS C-Tube(s) from the GentleMACS-Dissociator. Then, using a sterile transfer pipette or a 1,000 μl XL barrier pipette tip within a sterile laminar flow hood, add 6 ml of fresh pre-warmed (37°C) collagenase I digestion solution to each tube.
8. Incubate the minced kidney cortex samples at 37°C in a cell culture incubator (supplied w/ 5% CO_2_) with gentle orbital shaking for 15 min.
9. After 15 min, remove the samples from the incubator and place them back onto the GentleMACS-Dissociator. This time, run the preset program “m_brain_02.”
10. As before, remove the GentleMACS C-Tube(s) from the GentleMACS-Dissociator. Then, using a sterile transfer pipette or a 1,000 μl XL barrier pipette tip within a sterile laminar flow hood, add 2 ml of fresh pre-warmed (37°C) collagenase I digestion solution to each GentleMACS C-Tube to bring the final volume to 10 ml.
11. Return samples to the incubator and dissociate the tissues with gentle orbital shaking for an additional 15–20 min or until the tissue pieces are thoroughly digested.^∧∧^
  ° (^∧∧^Over/under digestion of the tissue pieces may result in a reduced cellular yield and/or viability; digestion times should be experimentally adjusted by the researcher accordingly, to ensure over/under digestion does not occur).
12. Following tissue digestion, transfer the cell suspensions from the GentleMACS C-Tube(s) into a 50 ml conical tube (or larger) and neutralize the collagenase I digestion solution with ∼40 ml of fresh ice-cold DMEM/F12 media.^**^
13. Next, centrifuge the cell suspensions at 200G for 3 min at 4°C.
14. Carefully remove the supernatant, gently resuspend the cell pellets in 10 ml of fresh ice-cold DMEM/F12^**^, and centrifuge the cell suspension again at 200G for 3 min at 4°C.
15. Make Percoll solution (25 ml/animal)^∧∧∧^:
° Note, the recipe listed below is for one sample; scale reagents according to the number of samples being processed. (^∧∧∧^This step can be done fresh or made up to 24 h prior to the cell isolation and stored at 4°C until needed.)
• 12.5 ml of ice-cold MEM/F-12, HEPES (15 mM) media (Gibco).
• 11.25 ml of Percoll-Plus solution (Cytiva).
• 562.5 μl of 20× PBS^–/–^ solution.
• 687.5 μl of filtered ultra-pure H_2_O.
16. Perform density gradient centrifugation to separate tubular and glomerular fraction as defined previously (18, 19):
• Resuspend cell pellets in 25 ml of 45% (vol/vol) sterile Percoll-Plus solution within a 50 ml conical tube.
• Centrifuge the cell suspensions at 5500G for 30 min at 4°C.
° (Note: centrifugation in this step should be performed without braking.)
17. Collect tubule fraction from the upper most layer of the Percoll solution (∼5 ml of total volume).
18. Wash tubular fractions with 20 ml of fresh ice-cold DMEM/F12 media.
19. Centrifuge the samples at 300G for 5 min at 4°C.
20. Remove the supernatant, resuspend the cell pellets in 7.5 ml of pre-warmed (37°C) Accutase solution (Sigma-Aldrich), and incubate samples again at 37°C in a cell culture incubator with gentle orbital shaking for 10–15 min (note: over digestion during this step can reduce overall cell viability/yield).
21. Neutralize the Accutase solution by adding 32.5 ml of ice-cold DMEM/F12 media^**^ to each cell suspension tube.
22. Then, filter the cell suspension through a 70-μm cell strainer followed by two 40-μm cell strainers to remove any large undigested cellular aggregates and glomeruli from the samples, respectively.
23. Centrifuge the final filtered cell suspension flow through at 300G for 5 min at 4°C.
° Remove the supernatant and resuspend the cell pellets with 5 ml of ice-cold microbead resuspension buffer (pH 7.2) (^#^see Table 1 for the exact solution compositions, considerations, and suppliers):
• 2 mM EDTA (29.224 mg).
• 0.5% BSA (0.25 g).
• 50 ml 1× PBS^–/–^.
° Note, the recipe listed above is to make 50 ml of microbead resuspension buffer; scale reagents according to the number of samples being processed. Typically, 400 ml of microbead resuspension buffer is sufficient for the processing of 4–6 independent samples.
° Note, be sure to sterile filter the entire microbead resuspension buffer solution by passing it through a 0.22-μm sterile filter prior to use. Additionally, we recommend degassing the microbead resuspension buffer to remove any air bubbles that might interfere with magnetic microbead conjugation or magnetic column separation.
24. Centrifuge the cell suspensions at 300G for 5 min at 4°C.
25. Remove the supernatant and resuspend the cell pellets in 500 μl of fresh ice-cold microbead resuspension buffer.
26. Split the cell suspension in half by transferring 250 μl into two separate 1.5 ml microcentrifuge tubes.
27. Next, add 5 μl of anti-mouse CD16/32 blocking antibody (Miltenyi Biotec FcR-blocking, 1:50) to each of the 1.5 ml microcentrifuge tubes containing the 250 μl of cell suspension.
28. Incubate the mixture by gently rotating tubes end-over-end at 4°C for 20 min.
29. After FcR-blocking is complete, add 8.5 μl of anti-CD326 (EPCAM) magnetic microbead solution (1:30) to each of the 1.5 ml microcentrifuge tubes.
30. Incubate the mixture by gently rotating tubes end-over-end at 4°C for 20 min.
31. After anti-CD326 magnetic microbead labeling is complete, wash the cell suspensions by added 1.2 ml of fresh ice-cold microbead resuspension buffer to each of the 1.5 ml microcentrifuge tubes.
32. Then, centrifuge cell suspensions at 300G for 10 min at 4°C.
33. Carefully, remove all the supernatant and gently resuspend the cell pellets in 250 μl of fresh ice-cold microbead resuspension buffer.
34. Recombine the contents of the two separated 1.5 ml microcentrifuge tubes, generated from the total cells of one individual mouse in step 26, to obtain a single cell solution with a final volume of ∼500 μl, and place the cells on ice.
35. Prepare LS columns for magnetic cell sorting by first placing the magnetic LS columns in an appropriate MACS magnet attached to a MACS magnetic stand.
36. After LS columns are properly positioned onto MACS magnets, place a 15 ml conical/collecting tube beneath each magnetic LS column.
37. Apply 3 ml of fresh ice-cold microbead resuspension buffer to each LS column and allow the solution to fully pass through the column to prime them for magnetic cell separation.
38. Once LS columns are primed, apply the total 500 μl suspension of magnetically labeled cells from one mouse to one magnetic LS column and allow cells to flow through the column entirely.
39. Next, wash cells within each LS column 4 times with 3 ml of ice-cold microbead resuspension buffer.
  ° Note, cells of interest will be contained within the column flow through (the negative fraction) in this step. Cells that are magnetically labeled in this step, and thus retained within the LS column (the positive fraction), contain CD326+ epithelial cells that might reduce the overall purity of isolated MRPECs and are subsequently discarded.
40. Place the LS column flow through tube (containing cells from the negative fraction) on ice and discard the LS columns containing the CD326+ epithelial cells.
41. Centrifuge the CD326− collection tubes at 300G for 10 min at 4°C.
42. Remove the supernatant and repeat steps 26 through 28 for the CD326- cell suspensions.
43. After FcR-blocking is complete, add 7.3 μl of anti-CD146 magnetic microbead solution (1:35) to each of the 1.5 ml microcentrifuge tubes.
44. Repeat steps 30 through 39 for the CD326− cell suspensions.
° Note: This time, cells of interest (CD326−/CD146+ MRPECs) are retained within the LS magnetic column (the positive fraction). The flow through obtained within the collecting tubes (the negative fraction) does not contain MRPECs and is subsequently discarded.
45. Remove LS columns from their magnetic stand and place them in a new collecting tube.
46. Then, add 5 ml of fresh ice-cold microbead resuspension buffer to the LS column.
47. Gently push the LS plunger into the top of the column to expel cells into the new collecting tube and obtain the desired CD326−/CD146+ cells.
48. This cell suspension is then run through a second cycle of LS column purification to ensure any remaining CD146− cells are thoroughly removed from the CD326−/CD146+ MRPEC containing fractions.
49. After the second LS column purification cycle, the flow through collecting tubes are discarded, and LS columns are removed from their magnetic stands and placed into new collecting tubes.
50. Add 5 ml of fresh pre-warmed (37°C) complete MV-EC specific media (Lonza EGM-MV2) to the top of each LS column, and gently push the LS plunger into the top of the column to expel the final purified CD326−/CD146+ MRPEC cells into the collecting tube.
51. Centrifuge the purified MRPEC cells at 300G for 10 min at 4°C.
52. Remove the supernatant and resuspend MRPEC cells in 1.5 ml of fresh pre-warmed (37°C) complete MV-EC specific media (Lonza EGM-MV2).
53. Lastly, seed total MRPECs obtained from one mouse onto a human plasma fibronectin (10 μg/ml/dish, PromoCell) precoated 35-mm Primaria cell culture dish, or 35-mm μ-Dish-high Ibidi imaging plate, and place it into a 37°C cell culture incubator (supplied w/ 5% CO_2_) for monoculture outgrowth.

**FIGURE 1:**
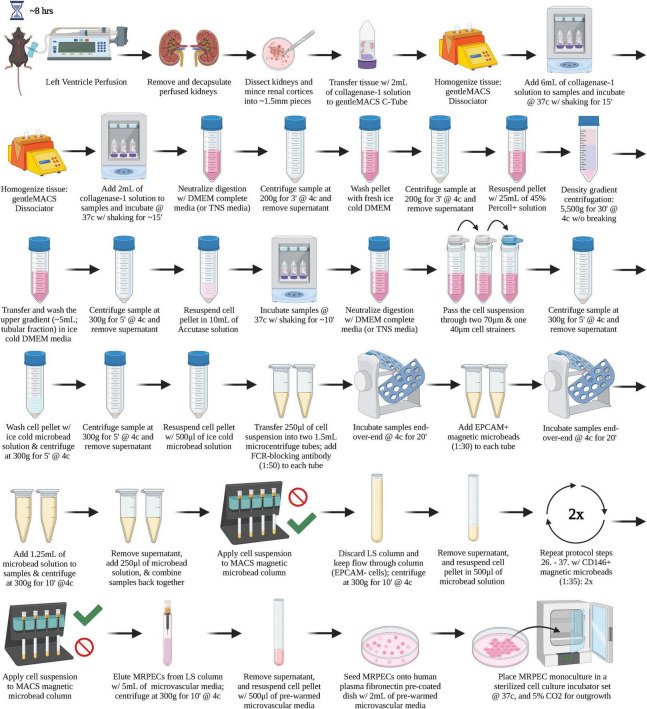
A graphical overview of the step-by-step procedure for the isolation and monoculture of primary MRPECs.

A graphical overview of the step-by-step procedure for the isolation and monoculture of primary MRPECs is shown in Figure 1.

### 3.2. Experimental methodologies

#### 3.2.1. Animals

Male C57BL/6J mice were purchased from The Jackson Laboratory (Bar Harbor, ME, USA) at 6–8 weeks of age, and were subsequently utilized to isolate primary MRPECs for all downstream *in vitro* monoculture experiments. All animal experiments were conducted in strict accordance with the recommendations, outlined within “The Guide for the Care and Use of Laboratory Animals,” curated by the National Institutes of Health (20). All protocols/procedures were approved by the Institutional Animal Care and Use Committee (IACUC) at The University of Arizona, and all precautions were made to minimize animal distress.

#### 3.2.2. MRPEC purification and isolation from mouse renal cortices

MRPECs were initially isolated utilizing a modified method described previously (19). The following modifications were incorporated as we were unable to obtain the purity of MRPECs suitable for downstream physiological and pharmacological studies (Supplementary Figure 1). Briefly, we altered the renal enzymatic tissue digestion buffers’ composition by replacing collagenase type 4 with collagenase type 1 and trypsin was replaced by accutase, respectively. Collagenase type 1 was selected as collagenase type 4 enzymes (e.g., MMP-9) were previously reported to facilitate phenotypic loss in primary renal peritubular endothelial cells (18), while accutase was employed to aid in the retention of EC specific markers that are known to be trypsin sensitive (e.g., CD31, VE-cadherin, etc.) (19, 21, 22). Additionally, GentleMACS-C tubes with automated tissue dissociation (Miltenyi Biotec) were employed to reduce kidney processing times and improve overall cell integrity. Cell suspensions were then subjected to one cycle of EPCAM+ (CD326) magnetic microbead (Miltenyi Biotec) depletion, *via* negative selection, to remove contaminating epithelial cells, as described previously (23). MRPECs were then purified and isolated *via* two positive selection cycles with CD146+ (MCAM) magnetic microbeads (Miltenyi Biotec). Twice purified MRPEC were subsequently seeded onto either human plasma fibronectin (10 μg/ml/dish, PromoCell) precoated 35-mm μ-Dish-high cell culture imaging dishes (Ibidi), and/or 35-mm Primaria cell culture dishes (Corning). Fibronectin coating was chosen as it was previously reported to regulate PLVAP (PV-1) localization to endothelial fenestrae by stabilizing microtubules (24).

#### 3.2.3. MRPEC monoculture

Purified MRPECs were first seeded onto human plasma fibronectin (10 μg/ml/dish, PromoCell) precoated 35-mm μ-Dish-high Ibidi imaging dishes or 35-mm Corning Primaria cell culture dishes and incubated overnight at 37°C with 5% CO_2_ in MV-EC specific media (EGM-MV2, Lonza) supplemented with 50 ng/ml of VEGF-165 (R&D Systems). The following day, cells were gently washed twice with pre-warmed (37°C) 1× PBS^–/–^ to remove cellular debris and non-adherent cells that remained prior to returning them to the incubator with 2 ml of fresh complete MV-EC specific media (EGM-MV2, Lonza). The media was replenished every 24 h thereafter. After 48 h, cells were transferred onto a sterile circular rotor within a 37°C cell culture incubator, set at ∼45 rpm. After 10 days in monoculture, MRPECs were assessed for purity and MV-EC phenotype retention.

#### 3.2.4. Phase-contrast microscopy of MRPEC monocultures

Periodically, throughout the 10 days of MRPEC monoculture, cellular morphology and outgrowth were monitored by phase-contrast microscopy. MRPEC cultures were monitored for the presence of any contaminating cell types, characteristic endothelial colony outgrowth, and for the appearance of typical EC cobblestone-like morphology upon maturation and contact inhibition. All phase-contrast micrographs of MRPEC monocultures were obtained by using an EVOS 4× fluorite LWD phase-contrast 0.13NA/10.58WD objective on the Invitrogen EVOS M5000 Cell Imaging System.

#### 3.2.5. Flow cytometry analysis of MRPEC monocultures

Proceeding purification, isolation, and 10 days of monoculture, purity of MRPEC monocultures were assessed by flow cytometry analysis. Initially, MRPEC culture media was removed from culture dishes, and cells were washed twice with pre-warmed (37°C) 1× DPBS^+/+^ to remove serum containing media. Then, 1 ml of pre-warmed (37°C) 1× Accutase solution (Sigma-Aldrich) was applied to the adherent MRPECs for 5–7 min to facilitate detachment. Cells were collected into a 15 ml conical tube containing 9 ml of PBS^–/–^ supplemented with 1% FCS and 2 mM EDTA. Cells were then pelleted at 300 g for 5 min at 4°C and counted using a TC20 Automated Cell Counter (Bio-Rad). Cells were then divided and strained into 5 ml Falcon round bottom polystyrene test tubes, with a 35 μm nylon mesh cell strainer snap cap at a concentration of 500,000 cells/tube. Cells were subsequently blocked with CD16/32 FcR-blocking antibody at a concentration of 1:50 for 20 min at 4°C, to mitigate non-specific binding. Then, cells were left unstained or stained with human anti-mouse CD146-PE+, CD31-BV515+, and CD144-APC+ recombinant monoclonal antibodies, or with recombinant human IgG PE+, BV515+, and APC+ conjugated isotype control antibodies, at a concentration of 1:50 for 20 min at 4°C, per manufacturer’s recommendation. Cells were then washed twice with 1 ml of PBS^–/–^ supplemented with 1% FCS and 2 mM EDTA (FACS Buffer), to remove unbound antibodies that remained. Following staining and washing steps, cells were resuspended in 350 μl of FACS Buffer. MRPECs were then analyzed by flow cytometry analysis on a BD FACSCanto II flow cytometer, to assess monoculture purity. (See Tables 1, 2 for further solution and antibody details.) Analyses and data figures were preformed/constructed using Flowjo V.10 software (BD). Data was cleaned in Flowjo (BD) using FlowAI/FlowClean plugins and normalized for batch analysis using the CytoNorm plugin with 10 cluster analysis iteration. All flow cytometry data was then analyzed by applying the same standard gating from unstained controls across all test samples. Between 32,000 and 52,000 high quality single cell events were then analyzed per sample in Flowjo (BD) utilizing their comparative histogram populations tool. Unstained and isotype control samples were both employed as comparators to assess MRPEC purity. Purity was based on Flowjos’ (BD) SE Dymax % positive cells, with 300 bins applied to further account for batch effects and machine acquisition variability/errors across our data sets. Chi-squared T(x) values ≥4 were considered statistical significance in our flow cytometry data, as described by Flowjo (BD).

#### 3.2.6. Immunofluorescence microscopy of MRPEC monocultures

After 10 days of monoculture, MRPEC cells were prepared for immunofluorescence microscopy. First, cells were washed twice with pre-warmed (37°C) 1× DPBS^+/+^ to remove cellular debris, and cell media components. Cells were subsequently fixed with 1 ml of Invitrogen Image-iT Fixative Solution (4% formaldehyde, methanol-free) for 10 min at room temperature. After 10 min, cells were washed three times with 1× DPBS^+/+^. Once fixed, cells were permeabilized in 1× DPBS^+/+^ containing 0.1% Triton X-100 for 10 min (for PLVAP and GSL-1 permeabilization, 1× DPBS^+/+^ containing 0.1% Triton X-100 was substituted for 10 μg/ml of digitonin in 1× DPBS^+/+^ to better preserve their architecture for IF staining). Cells were then washed with 1× DPBS^+/+^ three times before applying 400 μl of Image-iT FX Signal Enhancer for 30 min at room temperature to reduce the chance of non-specific antibody binding, per manufacturer’s recommendations. Cells were then blocked for 1 h at room temperature in BlockAid Blocking Solution (Invitrogen). After blocking, primary antibodies were diluted into BlockAid Blocking Solution at their respective dilutions (see Table 2 for a complete list of antibodies, dilutions, and supplies), and allowed to conjugate at room temperature for 1 h or at 4°C overnight. Primary antibody solutions were then removed, and cells were washed three times with 1× DPBS^+/+^ for 5 min each time. Secondary fluorescent antibodies were then diluted into BlockAid Blocking Solution at their respective dilutions (see Table 2 for a complete list of antibodies, dilutions, and suppliers), and allowed to conjugate to primary antibodies at room temperature for 1 h, in the dark protected from light. Cells were then washed four times with 1× DPBS^+/+^ for 5 min each time. Once cell staining was completed, Ibidi Mounting Medium+ DAPI was added drop-wise over the stained cells until covered. Cells were imaged 24 h thereafter, using an EVOS 20× fluorite LWD phase-contrast 0.45NA/6.12WD objective and/or an Olympus UPlanSApo infinity-corrected 40× objective on the Invitrogen EVOS M5000 Cell Imaging System. Images were then overlayed using FIJI (ImageJ) software.

#### 3.2.7. Capillary sprouting Matrigel assay of MRPEC monocultures

The capillary sprouting Matrigel assay used within this study was volume modified from the UPM Biomedical manufacturer’s technical product guide on, “Smooth muscle cells and endothelial cells: Comparative study of 3D culture in GrowDex & Matrigel,” to accommodate for the use of a 24 well plate-based system. The evening before the capillary sprouting Matrigel assay was conducted, the Matrigel solution (Corning) was placed in the fridge at 4°C on ice to thaw overnight. Additionally, the evening before we placed 24 well plates, 1.5 ml microcentrifuge tubes, and sterile barrier pipette tips in a −20°C freezer overnight for use during the assay preparation. The following day, media was removed from MRPEC culture dishes, and the cells were washed twice with pre-warmed (37°C) 1× DPBS^+/+^, to remove any remaining serum containing media. Then, 1 ml of pre-warmed (37°C) 1× Accutase solution (Sigma-Aldrich) was applied to the adherent MRPEC monocultures for 5–7 min to facilitate detachment from culture dishes. Cells were subsequently collected into a 15 ml conical tube containing 9 ml of complete MV-EC specific media (Lonza EGM-MV2) to neutralize the Accutase enzymatic activity. Cells were then pelleted at 300 g for 5 min at 4°C and counted using a TC20 Automated Cell Counter (Bio-Rad). Approximately 50,000 MRPEC cells were then blocked in 500 μl of BlockAid Blocking Solution for 30 min at room temperature, per manufacturer’s recommendations. Cells were then stained with 20 μg/ml of Alexa Fluor 488-Conjugated GSL-1 antibody suspended in 500 μl of BlockAid Blocking Solution for 30 min at room temperature, according to manufacturer’s recommendations. Stained MRPECs were then washed twice with 1 ml of 1× PBS^–/–^, to remove any remaining unbound GSL-1 staining solution. Following GSL-1 staining, 250 μl of ice-cold Matrigel was first added to each well of the 24 well plate. Then, in a pre-chilled 1.5 ml microcentrifuge tube, 125 μl of ice-cold Matrigel was diluted 1:1 with complete MV-EC specific media (Lonza EGM-MV2). Following 1:1 Matrigel dilution, 10 μl of cell suspension containing a total of approximately 1,000 cells/μl was mixed into a pre-chilled 1.5 ml microcentrifuge tube. This mixture was rapidly transferred to one well of the 24 well plate. This process was repeated until 4 total wells/condition were filled with a total of 510 μl/well of Matrigel embedded MRPEC stained with GSL-1. Then the entire 24 well plate was allowed to solidify at 37°C for 1 hour prior to the addition of 150 μl of complete MV-EC specific media (Lonza EGM-MV2) onto the top of each Matrigel containing well. The entire 24 well plate was then placed back into a 37°C cell culture incubator and allowed to grow for 36 h prior to imaging.

## 4. Results

### 4.1. Phase contrast micrographs of MRPEC monocultures

**FIGURE 2:**
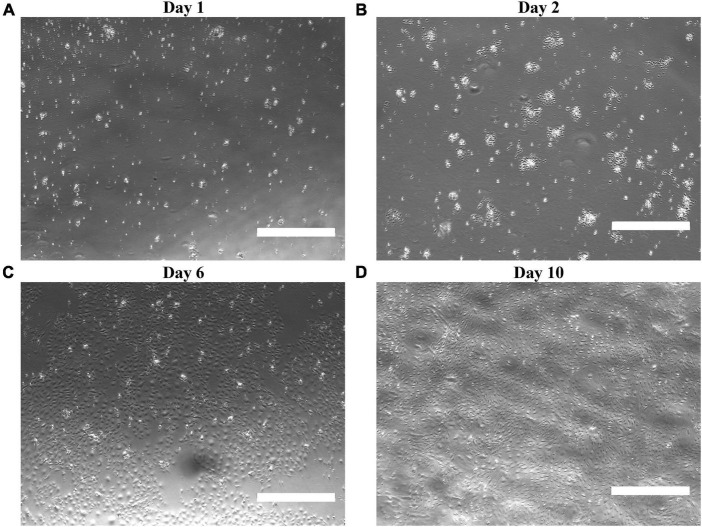
Characterization of isolated MRPECs outgrowth and morphology in monoculture. Representative phase-contrasted photomicrographs of MRPEC monoculture, outgrowth, and morphology at various post-isolation time points. MRPECs monoculture, outgrowth, and morphology at **(A)** 1 day post-isolation, **(B)** 2 days post-isolation, **(C)** 6 days post-isolation, and **(D)** 10 days post-isolation. *N* = 3–5/time point. Scale bars, 750 μm.

Proceeding MRPEC isolation, brightfield micrographs revealed that MRPEC monocultures seeded onto human plasma fibronectin precoated 35-mm Corning Primaria plates, or 35-mm μ-Dish-high Ibidi imaging plates, increased MRPEC seeding density, promoted uniform outgrowth, and preserved cell morphology over 10 days post-isolation (Figure 2). Typical MV-EC morphology and contact inhibition was observed at 6–10 days post isolation (Figures 2C, D).

### 4.2. Flow cytometry analysis of isolated MRPEC

Proceeding purification, isolation, and 10 days of monoculture, we first sought to assess the purity of MRPEC monocultures by utilizing flow cytometry analysis (see section “3.2. Experimental methodologies” and Table 2 for flow cytometry details and antibody concentrations used to assess primary MRPEC cultures purity and expression of phenotypic MRPEC biomarkers, respectively).

**FIGURE 3:**
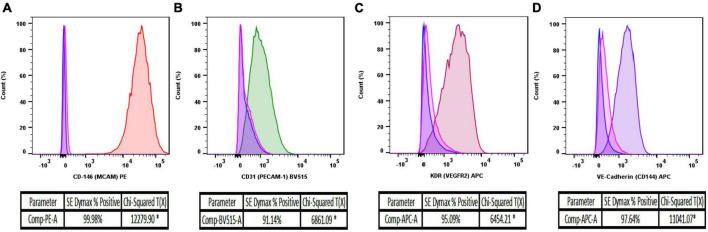
Flow cytometry analysis of isolated MRPEC monocultures 10 days post-isolation. Representative flow cytometry histograms of MRPEC monocultures 10 days post-isolation. **(A)** CD146-PE+ (red) MRPECs vs. IgG-PE+ isotype (pink) and unstained (blue) controls, **(B)** CD31-BV515+ (green) MRPECs vs. IgG-BV515+ isotype (pink) and unstained (blue) controls, **(C)** KDR (VEGFR2)-APC+ (burgundy) MRPECs vs. IgG-APC+ isotype (pink) and unstained (blue) controls, **(D)** VE-cadherin-APC+ (purple) MRPECs vs. IgG-APC+ isotype (pink) and unstained (blue) controls. *Chi-squared T(x) values ≥4 are considered statistically significant. *N* = 4–12 mice/marker.

BD FACS Canto-II flow cytometry analysis, 10 days after primary cell isolation and monoculture, revealed that MRPECs display an average CD146-PE+ purity of 99.9% compared to unstained and IgG isotype controls (Figure 3A). Furthermore, isolated MRPECs exhibited an average CD31-BV515+ purity of 90.8%, an average KDR-APC+ purity of 95.1%, and an average VE-cadherin-APC+ purity of 97.8% compared to unstained and IgG isotype controls (Figures 3B–D). Utilizing our refined MRPEC isolation method, we obtained a greater purity of CD31+ and CD146+ MRPECs 10 days post isolation compared to the previous unrefined method (19) by 90.12 and 21.75%, respectively (Figures 3A, B and Supplementary Figure 1).

### 4.3. Immunofluorescence analysis of isolated MRPEC

Following the results obtained by flow cytometry analysis, we next sought to confirm these results by leveraging immunofluorescence (IF) microscopy to probe primary MRPECs for known pan-EC biomarkers and specific phenotypic markers. As MRPECs have previously been reported to express the pan-EC markers CD31 and VE-cadherin, we utilized IF microscopy to confirm the presence and phenotypic retention of these pan-EC proteins on isolated MRPECs (25). See section “3.2. Experimental methodologies” and Table 2 for detailed IF preparation methods and antibody concentrations.

**FIGURE 4:**
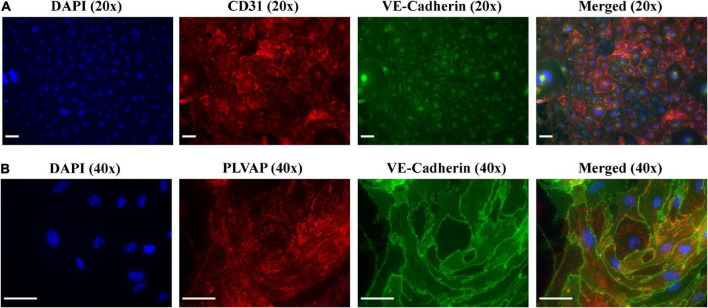
Immunofluorescent imaging (IF) and characterization of MRPECs 10 days post-isolation. Representative IF-photomicrographs of MRPEC monocultures 10 days post-isolation. **(A)** Nuclear staining DAPI (panel 1, blue), PECAM1+ staining (panel 2, red), VE-cadherin+ staining (panel 3, green), and merged (panel 4); all representative images in panel **(A)** were taken at 20× magnification. **(B)** Nuclear staining DAPI (panel 1, blue), PLVAP+ staining (panel 2, red), VE-cadherin+ staining (panel 3, green), and merged (panel 4); all representative images in panel **(B)** were taken at 40× magnification. All representative images were obtained on an EVOS-M5000 fluorescence microscope system and merged by overlay in FIJI (ImageJ) software. *N* = 4–6 isolations. Scale bars, 50 μm.

**FIGURE 5:**
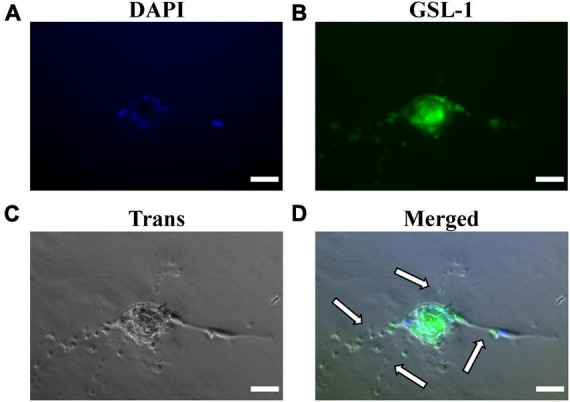
Matrigel embedded capillary sprouting capacity and Griffonia Simplicifolia Lectin-IB4 IF-staining of isolated MRPECs 10 days post-isolation. Representative phase-contrast photomicrographs and IF-images of MRPECs embedded in a Matrigel assays 36 h after Griffonia Simplicifolia Lectin-IB4 (GSL-1) staining and implantation. **(A)** Nuclear staining, DAPI (blue), **(B)** GSL-I staining (green), **(C)** phase-contrasted photomicrograph of embedded MRPECs (gray), and **(D)** overlay (merged); white arrows indicate capillary spouts/tubes. All representative images were taken at 20× magnification. All images were obtained on an EVOS-M5000 fluorescence microscope system and merged by overlay in FIJI (ImageJ) software. *N* = 4 isolations. Scale bars, 50 μm.

After 10 days of monoculture, IF-imaging of MRPECs revealed high expression levels of both CD31 and VE-cadherin (Figure 4A). To distinguish isolated cortical MRPECs from cortical glomerular capillary MV-ECs, which also express CD31 and VE-cadherin, cells were probed for the expression of MRPEC specific phenotypic marker plasmalemma vesicle associated protein (PLVAP), which is highly expressed by MRPECs but not by glomerular capillary MV-ECs (10, 26). Additionally, monocultures were probed for the glomerular EC specific marker, EHD3 (EH Domain Containing 3), as it has been previously noted that injured glomerular endothelial cells can undergo *de novo* production of PLVAP (27). MRPEC monocultures were subsequently found to be devoid of EHD3 expression (Supplementary Figure 2). To further discriminate these cells from large vessel ECs, we probed for the MRPEC phenotype marker Griffonia Simplicifolia Lectin-IB4 (GSL-1), which is expressed by MRPECs but absent from large vessel ECs (10, 26). After 10 days of monoculture, IF imaging of MRPECs revealed high expression levels of both PLVAP and GSL-1 (Figures 4B, 5B). Lastly, MRPEC monocultures were probed for the presence of other contaminating cell types such as EPCAM+ epithelial cells, NG2+ mural cells (e.g., pericytes, smooth muscle cells, etc.), and FSP-1+ fibroblasts/myofibroblasts. MRPEC monocultures were subsequently found to be devoid of EPCAM+, NG2+, and FSP-1+ cells (Supplementary Figure 2).

### 4.4. Matrigel embedded capillary spout assay and GSL-1 staining of isolated MRPEC

Upon confirming that isolated MRPEC express high levels of both pan-EC markers and the known peritubular MV-EC marker PLVAP, we performed a Matrigel embedded capillary MV-EC sprout capacity assay as well as GSL-1 IF-staining of primary MRPEC. See section “3.2. Experimental methodologies” and Table 2 for detailed Matrigel embedded capillary MV-EC sprout capacity assay preparation methods and GSL-1 staining. After 36 h, Matrigel embedded MRPECs were observed, *via* IF-imaging, to be GSL-1+ and formed capillary branches (Figure 5). Furthermore, while MRPECs appear to have a reduced angiogenic potential, compared to other MV-ECs (23), these cells were observed to form longer capillary branches with sizable tubular diameters (Figure 5D). These findings are in accord with previously published findings in primary human peritubular MV-ECs (23).

## 5. Discussion

Proceeding an AKI episode, peritubular microvascular rarefaction (PMR) has been observed in both human and animal studies. This PMR exacerbates renal injury and increases the likelihood of AKI recurrence as well as the development of other diseases such as chronic kidney disease and CVD. As the renal microvasculature (MV) is essential for oxygen and nutrient delivery to extravascular tissue during proper repair processes, a greater understanding of how these cells function under healthy and disease conditions may provide novel insight into druggable targets that blunt/prevent microvascular dysfunction/rarefaction. However, the renal microvasculature repair processes following injury, and the mechanism(s) by which neovascularization and/or a reduction in microvascular dysfunction/rarefaction may improve renal recovery, remains undetermined.

As there remains very limited published methods available for the successful isolation and monoculture of RPECs (19, 23, 28), especially in mice (19), we have developed this refined protocol to aid in the investigation of these cells regarding downstream physiological and pharmacological model-based microvasculature studies. In the current study, we present a refined method for the successful purification, isolation, and monoculture of MRPECs. Our approach provides MRPECs that display characteristic MV-EC outgrowth in monoculture (Figure 2), exhibit expression and retention of pan-EC markers [i.e., KDR (VEGFR2), PECAM1 (CD31), VE-cadherin (CD144), and CD146 (MCAM)], and retain RPEC specific phenotypic markers (i.e., PLVAP and GSL-1) 10 days post-isolation and monoculture (Figures 3–5) (10, 26). Furthermore, these cells were found to be devoid of EHD3+ glomerular endothelial cells, EPCAM+ epithelial cells, NG2+ mural cells, and FSP-1+ fibroblast/myofibroblast cells (Supplementary Figure 2).

Utilization of this method may provide insight into novel RPEC pathways and elucidate druggable targets for drug discovery initiatives aimed at the reduction/inhibition of RPEC dysfunction/rarefaction in various renal and cardiovascular related diseases/pathologies. Additionally, this protocol may aid others in various translational research areas such as 3D tissue engineering, renal pathophysiology, CVD, vascular development, renal microvascular EndMT, toxicology, nanomedicine, etc.

## Data availability statement

The original contributions presented in this study are included in this article/Supplementary material, further inquiries can be directed to the corresponding author.

## Ethics statement

The animal study was reviewed and approved by the Institutional Animal Care and Use Committee (IACUC) at The University of Arizona, and all precautions were made to minimize animal distress. All animal experiments were conducted in strict accordance with the recommendations, outlined within “The Guide for the Care and Use of Laboratory Animals,” curated by the National Institutes of Health (20).

## Author contributions

ADT and RGS conceived the protocol studies and wrote and edited the manuscript. ADT, JJ, and RGS aided in the experimental design and reviewed the manuscript. ADT and JJ preformed the experiments and analyzed the data. All authors contributed to the manuscript and approved the final submitted version.

## Supplementary material

Supplementary Figure 1 Mouse renal peritubular endothelial cells (MRPEC) isolation utilizing previously published method without refinements. Flow cytometry analysis and immunofluorescence imagining of isolated MRPEC monocultures 10 days post-isolation. Representative flow cytometry histograms of MRPEC monocultures 10 days post-isolation. **(A)** CD31-APC+ (blue) MRPECs vs. IgG-APC+ isotype control (gold). **(B)** CD146-PE+ (green) MRPECs vs. IgG-PE+ isotype control (blue). Representative IF-photomicrographs of MRPEC monocultures 10 days post-isolation. **(C)** Nuclear staining DAPI (panel 1, blue), CD31+ staining (panel 2, red), and merged (panel 3, overlay). **(D)** Nuclear staining DAPI (panel 1, blue), VE-cadherin+ staining (panel 2, red), and merged (panel 3, overlay). All representative images were obtained on an EVOS-M5000 fluorescence microscope system and merged by overlay in FIJI (ImageJ) software. *Chi-squared T(x) values ≥ 4 are considered statistically significant. *N* = 3 isolations/control. Scale bars, 50 μm.

Supplementary Figure 2 Immunofluorescent imaging (IF) and characterization of MRPECs 10 days post-isolation. Representative IF-photomicrographs of MRPEC monocultures 10 days post-isolation. **(A)** Nuclear staining DAPI (panel 1, blue), RFP negative control (panel 2, red), EDH3+ staining (panel 3, green), and merged (panel 4, overlay). **(B)** Nuclear staining DAPI (panel 1, blue), EPCAM+ staining (panel 2, red), GFP negative control (panel 3, green), and merged (panel 4, overlay). **(C)** Nuclear staining DAPI (panel 1, blue), NG2+ staining (panel 2, red), GFP negative control (panel 3, green), and merged (panel 4, overlay). **(D)** Nuclear staining DAPI (panel 1, blue), FSP-1+ staining (panel 2, red), GFP negative control (panel 3, green), and merged (panel 4, overlay). All representative images in panels **(A–D)** were taken at 20× magnification. All representative images were obtained on an EVOS-M5000 fluorescence microscope system and merged by overlay in FIJI (ImageJ) software. *N* = 3 isolations/control. Scale bars, 50 μm.
