# Supplementary figures and images for: A refined protocol for the isolation and monoculture of primary mouse renal peritubular endothelial cells
Source: Front Cardiovasc Med. 2023 Feb 9;10:1114726. doi: 10.3389/fcvm.2023.1114726 (PMC9948610; doi:10.3389/fcvm.2023.1114726)

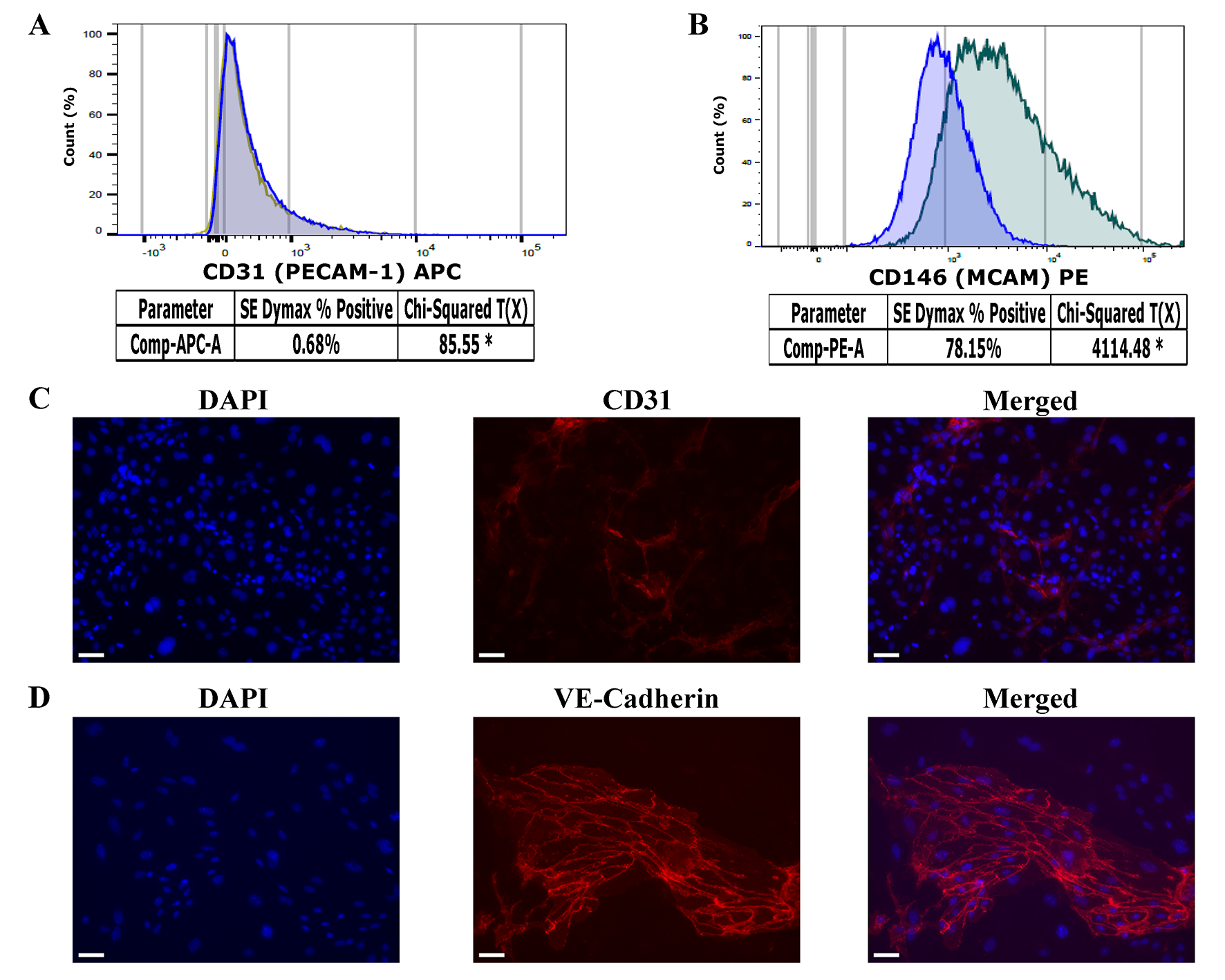

Supplement: Supplementary Figure 1 — Mouse renal peritubular endothelial cells (MRPEC) isolation utilizing previously published method without refinements. Flow cytometry analysis and immunofluorescence imagining of isolated MRPEC monocultures 10 days post-isolation. Representative flow cytometry histograms of MRPEC monocultures 10 days post-isolation. (A) CD31-APC+ (blue) MRPECs vs. IgG-APC+ isotype control (gold). (B) CD146-PE+ (green) MRPECs vs. IgG-PE+ isotype control (blue). Representative IF-photomicrographs of MRPEC monocultures 10 days post-isolation. (C) Nuclear staining DAPI (panel 1, blue), CD31+ staining (panel 2, red), and merged (panel 3, overlay). (D) Nuclear staining DAPI (panel 1, blue), VE-cadherin+ staining (panel 2, red), and merged (panel 3, overlay). All representative images were obtained on an EVOS-M5000 fluorescence microscope system and merged by overlay in FIJI (ImageJ) software. *Chi-squared T(x) values ≥ 4 are considered statistically significant. N = 3 isolations/control. Scale bars, 50 μm. [file Image_1.TIFF]

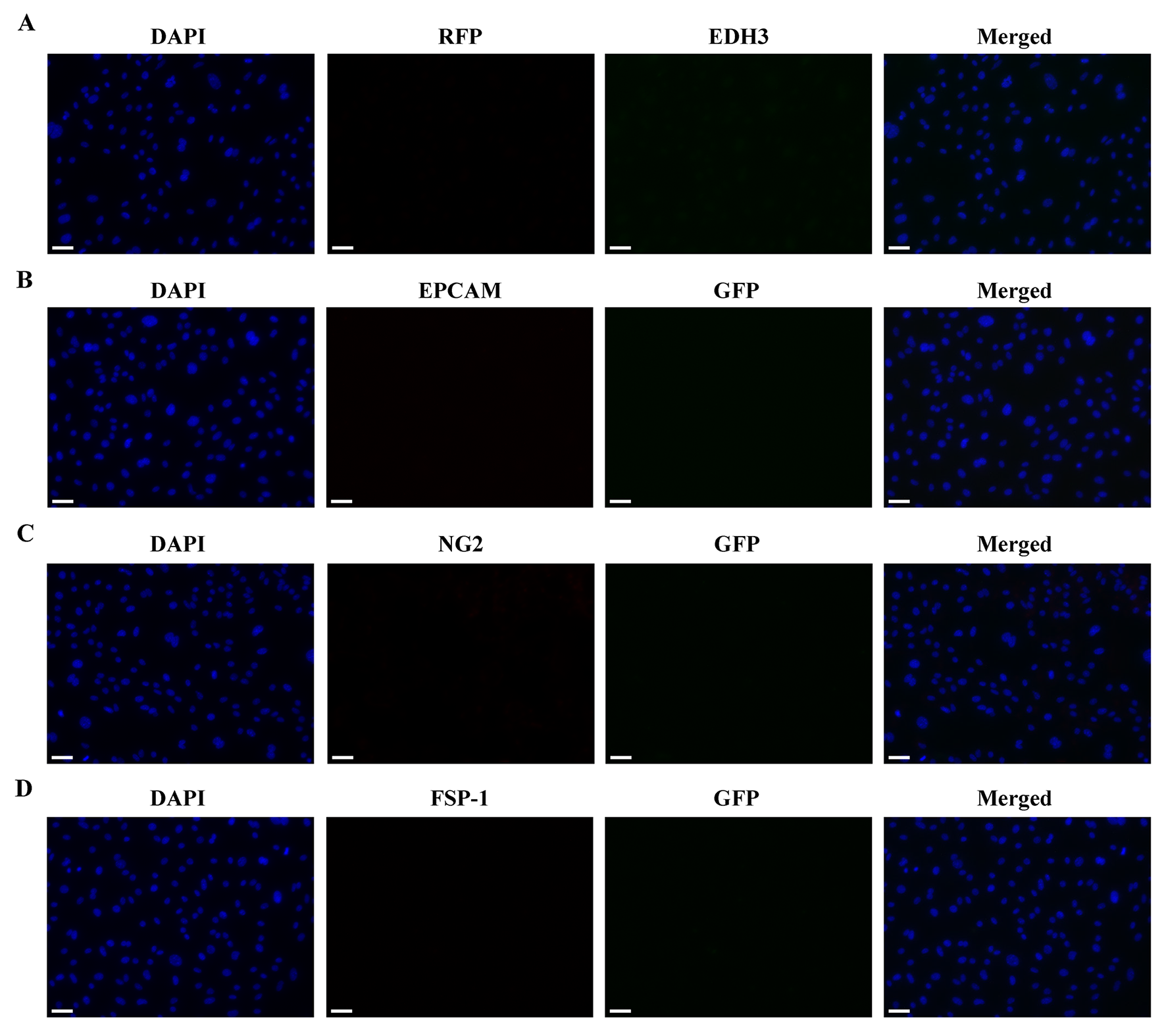

Supplement: Supplementary Figure 2 — Immunofluorescent imaging (IF) and characterization of MRPECs 10 days post-isolation. Representative IF-photomicrographs of MRPEC monocultures 10 days post-isolation. (A) Nuclear staining DAPI (panel 1, blue), RFP negative control (panel 2, red), EDH3+ staining (panel 3, green), and merged (panel 4, overlay). (B) Nuclear staining DAPI (panel 1, blue), EPCAM+ staining (panel 2, red), GFP negative control (panel 3, green), and merged (panel 4, overlay). (C) Nuclear staining DAPI (panel 1, blue), NG2+ staining (panel 2, red), GFP negative control (panel 3, green), and merged (panel 4, overlay). (D) Nuclear staining DAPI (panel 1, blue), FSP-1+ staining (panel 2, red), GFP negative control (panel 3, green), and merged (panel 4, overlay). All representative images in panels (A–D) were taken at 20× magnification. All representative images were obtained on an EVOS-M5000 fluorescence microscope system and merged by overlay in FIJI (ImageJ) software. N = 3 isolations/control. Scale bars, 50 μm. [file Image_2.TIFF]
